# The Phloem Intercalated With Xylem-Correlated 3 Receptor-Like Kinase Constitutively Interacts With Brassinosteroid Insensitive 1-Associated Receptor Kinase 1 and Is Involved in Vascular Development in *Arabidopsis*

**DOI:** 10.3389/fpls.2021.706633

**Published:** 2022-01-11

**Authors:** Ke Xu, Joris Jourquin, Maria Fransiska Njo, Long Nguyen, Tom Beeckman, Ana Ibis Fernandez

**Affiliations:** ^1^Department of Plant Biotechnology and Bioinformatics, Ghent University, Ghent, Belgium; ^2^VIB Center for Plant Systems Biology, Ghent, Belgium; ^3^Screening Core, VIB, Ghent, Belgium; ^4^Centre for Bioassay Development and Screening (C-BIOS), Ghent University, Ghent, Belgium

**Keywords:** LRR-RLK, BAK1, PXC3, CLE41, PXY, vascular development

## Abstract

Leucine-rich repeat receptor-like kinases (LRR-RLKs) play fundamental roles in cell-to-cell and plant-environment communication. LRR-RLKs can function as receptors perceiving endogenous or external ligands, or as coreceptors, which stabilize the complex, and enhance transduction of the intracellular signal. The LRR-RLK BAK1 is a coreceptor for different developmental and immunity pathways. In this article, we identified PXY-CORRELATED 3 (PXC3) as a BAK1-interacting LRR-RLK, which was previously reported to be transcribed in vascular tissues co-expressed with PHLOEM INTERCALATED WITH XYLEM (PXY), the receptor of the TDIF/CLE41 peptide. Characterization of *pxc3* loss-of-function mutants revealed reduced hypocotyl stele width and vascular cells compared to wild type, indicating that PXC3 plays a role in the vascular development in *Arabidopsis*. Furthermore, our data suggest that PXC3 might function as a positive regulator of the CLE41/TDIF–TDR/PXY signaling pathway.

## Introduction

Plants are sessile multicellular organisms and to perceive developmental cues and environmental stimuli, they rely on numerous sensory proteins on their cell surfaces. Receptor-like kinases (RLKs), one of the most important sensory protein groups in plants, play fundamental roles in cell-to-cell and plant–environment communication (van der Burgh and Joosten, 2019; Xi et al., 2019; Ou et al., 2021). The largest group of RLKs in plants have an extracellular leucine-rich repeat (LRR) domain and are therefore called LRR-RLKs. The *Arabidopsis* genome encodes more than 200 LRR-RLKs, which can be further grouped into 13 subfamilies (Shiu and Bleecker, 2001, 2003). Many of these LRR-RLKs function as receptors for phytohormones, endogenous peptides, and pathogen-derived molecules, regulating plant growth and defense responses (De Smet et al., 2009; Couto and Zipfel, 2016; He et al., 2018). LRR-RLKs sensing ligand signals in plants include BRASSINOSTEROID INSENSITIVE 1 (BRI1) for the phytohormone brassinosteroid (Li and Chory, 1997), PHYTOSULFOKINE RECEPTOR (PSKR) for endogenous PHYTOSULFOKINE peptides (Matsubayashi et al., 2002), FLAGELLIN SENSING 2 (FLS2) for the pathogen-derived flagellin (Gomez-Gomez and Boller, 2000; Chinchilla et al., 2006), and many more.

Somatic embryogenesis receptor kinases (SERKs), belonging to LRR-RLK subfamily II, have been extensively shown to serve as coreceptors for various LRR-RLK signaling pathways, in both plant growth and immunity pathways (Ma et al., 2016; He et al., 2018). SERK3, also called BRI1-ASSOCIATED RECEPTOR KINASE 1 (BAK1), is the best-studied ones among the five SERK family members in *Arabidopsis*. BAK1 was initially found to be involved in brassinosteroid perception (Li et al., 2002), but recent studies have shown diverse functions for BAK1 in the regulation of plant growth and immune responses by forming ligand-induced complexes with different LRR-RLKs. For example, BAK1 dimerizes with RGF1 INSENSITIVE receptors to regulate root development (Ou et al., 2016; Song et al., 2016), with HAESA and HAESA-LIKE 2 to control cell separation during floral organ abscission (Meng et al., 2016; Santiago et al., 2016), with ERECTA and ERECTA-LIKE 1 to regulate stomatal development (Meng et al., 2015), and with PSKR1 to regulate cell proliferation and elongation (Ladwig et al., 2015; Wang et al., 2015). Furthermore, BAK1 participates in plant immune responses by interacting with receptors, like FLS2 (Chinchilla et al., 2007), ELONGATION FACTOR-TU RECEPTOR (Roux et al., 2011), and PEP1 RECEPTOR 1 (PEPR1) (Tang et al., 2015). In many cases, although not all, dimerization of the ligand-binding receptor with SERK coreceptors is induced by the ligand. In addition to the ligand-induced receptor–coreceptor interactions, recent studies revealed the occurrence of ligand-free constitutive interactions between different LRR-RLKs (Smakowska-Luzan et al., 2018). The BAK1-INTERACTING RECEPTOR-LIKE KINASE 2 (BIR2) and BIR3 have been shown to constitutively interact with BAK1 and act as negative regulators of FLS2-BAK1 and/or BRI1–BAK1 complex formation (Halter et al., 2014; Imkampe et al., 2017). Also, the NUCLEAR SHUTTLE PROTEIN-INTERACTING KINASE 1 (NIK1) (Li et al., 2019), FLS2-INTERACTING RECEPTOR (FIR) (Smakowska-Luzan et al., 2018), and IMPAIRED OOMYCETE SUSCEPTIBILITY 1 (IOS1) (Yeh et al., 2016) constitutively interact with BAK1 or the ligand-sensing receptor. These ligand-free interactions have been shown often to regulate receptor–coreceptor complex formation.

BAK1, as well as SERK1 and 2, were also shown to be involved in vascular development by serving as coreceptors for TDIF RECEPTOR (TDR)/PHLOEM INTERCALATED WITH XYLEM (PXY), which senses the TRACHEARY ELEMENT DIFFERENTIATION FACTOR (TDIF) peptide (Fisher and Turner, 2007; Hirakawa et al., 2008), encoded by both the *CLE41* and *CLE44* genes (Ito et al., 2006). The SERK1-3 and PXY ectodomains were shown to interact in the presence of TDIF and, accordingly, the triple loss-of-function (*lof*) mutant *serk1-1serk2-1bak1-5* is insensitive to TDIF peptide treatments and shows defects in procambial cell organization (Zhang et al., 2016). Activation of the TDIF–TDR/PXY pathway results in upregulation of two *WUSCHEL RELATED HOMEOBOX (WOX)* genes, *WOX4*, and *WOX14* (Etchells and Turner, 2010; Hirakawa et al., 2010; Etchells et al., 2013). WOX14 induces the expression of another transcription factor, *TARGET OF MONOPTEROS 6 (TMO6)*. WOX14 and TMO6 in turn, stimulate the expression of *LATERAL ORGAN BOUNDARIES DOMAIN 4 (LBD4)* at the procambium–phloem boundary to control vascular stem cell proliferation (Smit et al., 2020).

In a previous study, three LRR-RLKs were identified and related to TDR/PXY *via in silico* co-expression and functional clustering analyses. These RLKs were named *PXY-CORRELATED (PXC) 1* to *3*, and PXC1 was indeed found to regulate vascular development by controlling secondary cell wall formation (Wang et al., 2013). Recent reports have shed light on the function of PXC2 (Goff and Van Norman, 2021). However, the function of *PXC3* remains unknown. In this article, we identified PXC3 as a BAK1 interactor and revealed that it functions in vascular development in a similar manner as TDR/PXY.

## Materials and Methods

### Growing Conditions and Plant Material

*Arabidopsis thaliana* (Col 0) seedlings were sown on solid half MS medium (Duchefa Biochemie B.V.) supplemented with 1% sucrose (VWR), 0.1 g l^–1^ Myo-inositol (Sigma–Aldrich), 0.5 g l^–1^ 2-(*N*-morpholino)ethanesulfonic acid (MES) (Duchefa Biochemie B.V.), and 0.8% Plant Tissue Culture Agar (Lab M, MC029). The plates were stratified for 2 days at 4°C and grown at 21°C under continuous light conditions.

Entry clones carrying the LRR-RLKs in pDONR/zeo (Invitrogen) were ordered from the Arabidopsis Biological Resource Center (ABRC): N4G33430ZEF (BAK1), N5G48380ZEF (BIR1), N3G28450ZEF (BIR2), N1G27190ZEF (BIR3), N2G41820ZEF (PXC3), N4G08850ZEF (MIK2), and N3G14840ZEF (LIK1). Receptor entry clones were combined with the pEN-L4-4-R1 (Karimi et al., 2007), carrying the cauliflower mosaic virus (*CaMV*) *35S* promoter, and pEN-R2-n/cGFP-L3 (Boruc et al., 2010), containing the N- or C- terminal half of the GFP, into the pB7m34GW destination vector by Gateway LR recombination reaction, to generate the corresponding *35Spro:LRR-RLK-n/cGFP* expression clones that were used for infiltration of *Nicotiana benthamiana* leaves. BAK1 was fused similarly with a 3× HA tag (pEN-R2-3 × HA-L3) (Van Leene et al., 2007). The S2G41820HGF clone containing the *PXC3* coding sequence with a C-terminal GFP fusion driven by the *CaMV 35S* promoter was also ordered from ABRC.

The *pxc3-1* (SALK_121365) and *pxc3-2* (SALK_092805) mutants were requested from the Nottingham Arabidopsis Stock Centre (NASC). The *pxc2-1* (SALK_055351C) and *pxc2-2* (SALK_018730C) mutants (also called *canar-1* and *canar-2*, respectively) were described previously (Hajny et al., 2020) and were kindly donated by Jiří Friml. The *pxy* mutant has been reported (Etchells and Turner, 2010) and was kindly donated by Eliana Mor and Bert De Rybel. The *pxypxc3-2* double mutant was obtained by crossing. The *BAK1pro:BAK1-GFP/bak1-4* has been reported (Ntoukakis et al., 2011) and was kindly donated by Ciryl Zipfel.

### Microsomal Extraction From *Arabidopsis thaliana* Seedlings

Microsomal extraction was performed according to Abas and Luschnig (2010) with modifications. *BAK1pro:BAK1-GFP/bak1-4/iGLV6* homozygous seedlings were germinated in a 2,000 ml Erlenmeyer containing 600 ml of liquid half MS medium. Five days after germination (DAG), mock or estradiol (2 μM) was added to the medium and incubated for 24 h. Then the growing medium was discarded and plant tissue was cross-linked on ice-cold 1% formaldehyde in PBS pH 7.4 for 30 min, then quenched with 300 mM glycine in cold PBS for 30 min and washed three times for 10 min with cold PBS. Four grams of tissue were weighed and frozen in liquid N_2_. Tissue was ground with a mortar and 1.5 ml of 1.4× extraction buffer [EB: 50 mM Tris pH 7.5, 25% D-Sorbitol, 10 mM Ethylenediaminetetraacetic acid (EDTA), 10 mM Ethylene glycol-bis (2-aminoethylether)-*N,N,N’,N’*-tetraacetic acid (EGTA), 50 mM sodium fluoride, 40 mM β-glycerol phosphate, 1mM sodium molybdate, 1 mM dithiothreitol (DTT), 1 mM phenylmethylsulfonyl fluoride (PMSF), and 1× cOmplete ULTRA Tablets (5892791001; Roche)] per gram of tissue was added. The samples were transferred to a 50 ml falcon tube, vortexed, then transferred to another tube containing high molecular weight insoluble polyvinylpolypyrrolidone (PVPP, 50 mg per gram of tissue) that had been previously equilibrated with EB, vortexed, and spun down. After transfer to the PVPP pellet, samples were vortexed, incubated on ice for 5 min, then centrifuged at 600 *g* for 5 min at 4°C. The supernatant was transferred to another tube and the pellet was extracted again twice with half and one-third of the initial EB volume. All supernatants were pooled. Finally, the pellet was centrifuged at 2,000 *g* for 5 min, the supernatant was added to the previous supernatant pool and the pellet was discarded. Pooled supernatants were vortexed and centrifuged at 6,000 *g* for 5 min then filtered through a mesh (Miracloth) to eliminate the remaining debris. Then an equal volume of water was added and samples were vortexed and centrifuged at 100,000 *g* for 2 h in a Beckman Coulter ultracentrifuge (Beckman Coulter L8-70). The microsomal pellet was washed with 10 mM Tris pH 7.3 and resuspended by pipetting in 1 ml of resuspension buffer [10 mM Tris pH 7.3, 150 mM NaCl, 1 mM EDTA, 10% glycerol, 1% Triton X-100, 1 mM PMSF, 20 mM NaF and 1× cOmplete ULTRA Tablets (Roche)]. The sample was transferred to a 2 ml eppendorf and centrifuged on a bench centrifuge at 21,000 *g* for 30 min at 4°C (Eppendorf Centrifuge 5427 R). The supernatant was transferred to a new tube and total protein was quantified with the Bradford assay.

### Affinity Purification and Sample Preparation

Two milligrams of total protein from microsomal extractions were incubated with 50 μl of anti-GFP μMACS MicroBeads (Miltenyi Biotec) for 1 h at 4°C. Beads were captured into μMACS columns (Miltenyi Biotec) according to the manufacturer’s instructions and washed four times with microsomal resuspension buffer. Then rinsed twice with 500 μl ABC Buffer [50 mM NH_4_HCO_3_ in H_2_O] to remove all the detergent. The column was removed from the magnet and immediately placed into a 0.5 mL low-bind eppendorf tube. Fifty microliters of ABC Buffer pre-heated at 95°C was added to the column to elute the beads.

For MS analysis, on-bead digestion, and sample preparation were performed as previously described (Wendrich et al., 2017), with the exception that the initial DTT and iodoacetamide treatments were performed for 1 h instead of 2 h.

### Mass Spectrometry and Data Analysis

The obtained peptide mixtures were introduced into an LC-MS/MS system, the Ultimate 3000 RSLC nano (Dionex, Amsterdam, Netherlands) in-line connected to an LTQ Orbitrap Velos (Thermo Fisher Scientific, Bremen, Germany). The sample mixture was loaded on a trapping column (made in-house, 100 μm internal diameter (I.D.) × 20 mm (length), 5 μm C18 Reprosil-HD beads (Dr. Maisch GmbH, Ammerbuch-Entringen, Germany). After back-flushing from the trapping column, the sample was loaded on a reverse-phase column (made in-house, 75 μm I.D. x 150 mm, 5 μm C18 Reprosil-HD beads, Dr. Maisch). Peptides were loaded with solvent A (0.1% trifluoroacetic acid, 2% acetonitrile), and separated with a linear gradient from 2% solvent A’ (0.1% formic acid) to 50% solvent B’ (0.1% formic acid and 80% acetonitrile) at a flow rate of 300 nl/min, followed by a wash step reaching 100% solvent B’.

The mass spectrometer was operated in data-dependent mode, automatically switching between MS and MS/MS acquisition for the 10 most abundant peaks in a given MS spectrum. In the LTQ-Orbitrap Velos, full scan MS spectra were acquired in the Orbitrap at a target value of 1E6 with a resolution of 60,000. The 10 most intense ions were then isolated for fragmentation in the linear ion trap, with a dynamic exclusion of 20 s. Peptides were fragmented after filling the ion trap at a target value of 1E4 ion counts.

The raw files were processed using the MaxQuant software (version 1.5.1.2) using standard settings as described in Wendrich et al. (2017).

### *Nicotiana benthamiana* Leaf Infiltration

The constructs for bimolecular fluorescence complementation (BIFC) and Co-IP were transformed into *A. tumefaciens* strain C58C1 by electroporation. *A. tumefaciens* cultures carrying receptor fusions to tags or the P19 suppressor (Scholthof, 2006), were diluted with infiltration buffer (10 mM MgCl_2_, 10 mM MES pH 5.7, and 100 μM acetosyringone) to obtain OD_600_ = 1. Then equal amounts of each culture were mixed and used for infiltration. Three days after infiltration, *N. benthamiana* leaves were imaged for BIFC or harvested in liquid N_2_ for Co-IP. Receptor interactions were compared only when they were infiltrated in the same leaves.

### Bimolecular Fluorescence Complementation

For BIFC assays, five leaves were infiltrated each time and five images were taken and averaged for each receptor pair combination per leaf with a Zeiss LSM 710 confocal microscope. GFP was excited at 488 nm and acquired at 493–542 nm. Quantification of the GFP signal in each image was performed with ImageJ using the mean gray value function.

### Protein Extraction and Western Blot

For Co-IP, frozen tobacco leaves were ground in liquid N_2_ and the extraction buffer [50 mM Tris pH 7.5, 150 mM NaCl, 1 mM EDTA, 10% (v/v) glycerol, 1% (v/v) Triton X-100, 1 mM PMSF, and 1× complete ULTRA Tablets (5892791001, Roche)] was added at the ratio of 2 μl per mg tissue (1:2 w:v). Samples were vortexed and centrifuged at 10,000 *g* for 15 min. The supernatant was collected and then filtered through a mesh (Miracloth) to eliminate the remaining debris. Protein concentration was quantified using the Qubit protein assay kit (Thermo Fischer Scientific). Approximately, 200 mg protein was used for Co-IP with GFP-trap magnetic agarose beads (gtma, Chromotek) for 1 h at 4°C with gentle shaking. The beads were collected and washed three times with washing buffer (10 mM Tris pH 7.5, 150 mM NaCl, 0.05% NP-40, and 0.5 mM EDTA). Bound proteins were eluted by boiling in Laemli sample buffer. Proteins were loaded on the gel and analyzed by Western blot with anti-HA antibody (1/2,000, 26183, Thermo Fisher Scientific), followed by secondary horseradish peroxidase (HRP)-conjugated anti-mouse antibody (1/10,000, NA931, Amersham Biosciences). Membranes were stripped with stripping buffer [1:1 (v:v) 100 mM Glycine-HCl pH 2.5 and 10% SDS buffer], and reblotted with anti-GFP antibody (1/5,000, ab290, Abcam), followed by secondary HRP-conjugated anti-rabbit antibody (1/10,000, NA934V, GE Healthcare).

For BAK1 protein level analysis in *Arabidopsis*, 30 seedlings at 5 DAG were harvested, and protein extraction and quantification were done as described above using the extraction buffer [50 mM Tris pH 7.5, 100 mM NaCl, 10% glycerol, 0.5% Triton X-100, 1 mM PMSF, 1 mM EDTA and 1× cOmplete ULTRA Tablets (5892791001; Roche)]. Samples were centrifuged for 30 min at 13,000 *g*, and 20 μg of total protein from the supernatant was loaded on the gel and blotted with anti-BAK1 antibody (1/5,000, AS12, Agrisera). Membranes were stripped with stripping buffer and reblotted with anti-tubulin antibody (1/2,000, T6199, Sigma). BAK1 signal in blots was quantified with ImageLab (Bio-Rad) 6.0 using the relative quantity function.

### Hypocotyl Stele Width Measurement

Three DAG seedlings of wild type, *pxy*, *pxc2-1*, *pxc2-2*, *pxc3-1*, *pxc3-2*, and *pxypxc3-2* were transferred from solid to liquid half MS medium with or without 5 μM of synthetic TDIF peptide (His-Glu-Val-Hyp-Ser-Gly-Hyp-Asn-Pro-Ile-Ser-Asn) (GenScript) and grown with gentle shaking for 4 days. Samples were then harvested and fixed with 4% (w/v) paraformaldehyde for 1 h under vacuum, washed twice with PBS, and then transferred into ClearSee solution [10% (w/v) xylitol, 15% (w/v) sodium deoxycholate, and 25% (w/v) urea] (Kurihara et al., 2015). After 3 days, cell walls were stained with 100 μg/ml calcofluor white in ClearSee solution for 1 h under vacuum and washed with ClearSee solution for 30 min. The stele width was measured in images obtained with a Zeiss LSM 710 confocal microscope. Calcofluor white was excited at 405 nm and acquired at 410–524 nm.

### Cross-Sections

For anatomical analysis of hypocotyl vascular development in *Arabidopsis* seedlings, cross-sections were made as described (Beeckman and Viane, 2000; De Smet et al., 2004). Briefly, 7 DAG seedlings were harvested and fixed by FAA solution (4% formaldehyde, 5% glacial acetic acid, and 50% ethanol) for 2 days and washed twice with phosphate buffer. Dehydration was performed using 30, 50, 70, 85, and 96% ethanol. Resin infiltration was performed using 30, 50, 70, and 100% Technovit 7100 (VWR, HKUL64709003). Two-step embedding was used and 5μm sections were cut with a microtome (Reichert Jung 2040). Sections stained with toluidine blue and images were taken with an Olympus BX53 DIC microscope using 500× magnification.

### qPCR Analysis

*PXC3* expression levels in *pxc3* mutants were determined in 7 DAG seedlings of wild type, *pxc3-1*, and *pxc3-2*. To test the *WOX4* and *WOX14* expression, 3 DAG seedlings of wild type, *pxy*, *pxc2-1*, *pxc2-2*, *pxc3-1*, *pxc3-2*, and *pxypxc3-2* were transferred from solid half MS medium to liquid half MS medium for 4 days with shaking. A 5 μM of TDIF peptide or mock was added to the samples for 8 h. Samples were harvested and frozen. RNA was extracted with the ReliaPrep RNA Miniprep System (Promega). First-strand cDNA was synthesized using the qScript cDNA Supermix (Quantabio). qPCR was performed using SyberGreen (Roche) and LightCycler real-time thermocycler (Roche). *ACT2* and *TUA4* were used as reference transcripts. Primers are listed in Supplementary Table 2.

### Statistical Analysis

To compare the mean fluorescence intensity from BIFC analysis and the hypocotyl stele width, a one-way ANOVA with multiple comparison analysis followed by Tukey’s test was performed using Graphpad Prism 8. For comparison of the TDIF treatment effect on the stele width of wild type and mutants, a linear model was fitted to the data using the genotype, the treatment and their interaction as explanatory variables. A two-way ANOVA was performed, followed by post hoc comparison of contrasts between the wild type and each mutant genotype using a Dunnett’s correction. The analysis was performed in R-4.0.3 using the “Car” and “Emmeans” packages. To compare the effect of TDIF peptide treatment on *WOX4* and *WOX14* expression between the different genotypes, peptide/mock transcript fold change was calculated and converted to Log2. Statistical differences between genotypes were then determined with one-way ANOVA followed by a post hoc comparison Tukey’s test using Graphpad Prism 8. For comparison of the relative BAK1 protein level, a one-way ANOVA with multiple comparison analysis followed by Tukey’s test was performed in Graphpad Prism 8.

## Results

### Phloem Intercalated With Xylem-Correlated 3 Constitutively Interacts With Brassinosteroid Insensitive 1-Associated Receptor Kinase 1

We have previously identified GLV6, a signaling peptide from the GOLVEN/Root meristem Growth Factor/CLE-like family, as a regulator of lateral root development (Fernandez et al., 2015). Considering that the *bak1-4* mutant partially suppressed the GLV6 phenotype and could be a coreceptor for the GLV pathway during lateral root development (data not shown) we designed an experiment to identify BAK1 interactors upon induction of the pathway. For this, we crossed an estradiol-inducible *GLV6* overexpression line (*iGLV6*) (Fernandez et al., 2020) with a reported *BAK1pro:BAK1-GFP/bak1-4* line (Ntoukakis et al., 2011) and performed affinity purification mass spectrometry (AP-MS) experiments in microsomal-enriched fractions using anti-GFP beads after estradiol induction or mock. Unfortunately, statistical analysis of estradiol-treated *vs.* mock seedlings did not reveal significant differences, which could be because BAK1 is a coreceptor for different pathways resulting in diluted output (Supplementary Table 1). We remarked that regardless of the treatment, known BAK1 interactors were pulled down, as would be expected. These included peptides from the LRR-RLKs BIR1, BIR2, and BIR3, which have been shown to constitutively interact with BAK1 and negatively regulate its complex formation with ligand-binding receptors (Gao et al., 2009; Halter et al., 2014; Imkampe et al., 2017). In addition, several peptides for three other LRR-RLKs; PXC3, MALE DISCOVERER 1 (MDIS1)/INTERACTING RECEPTOR LIKE KINASE2 (MIK2), and LYSM RLK1-INTERACTING KINASE 1 (LIK1) were recovered (Supplementary Table 1). Interestingly, in a similar experiment where a SERK1-CFP fusion was used as a bait, five of these receptors coeluted with SERK1, and four of them (BIR2, BIR3, LIK1, and PXC3) were significantly enriched compared to the control (Smaczniak et al., 2012).

**FIGURE 1:**
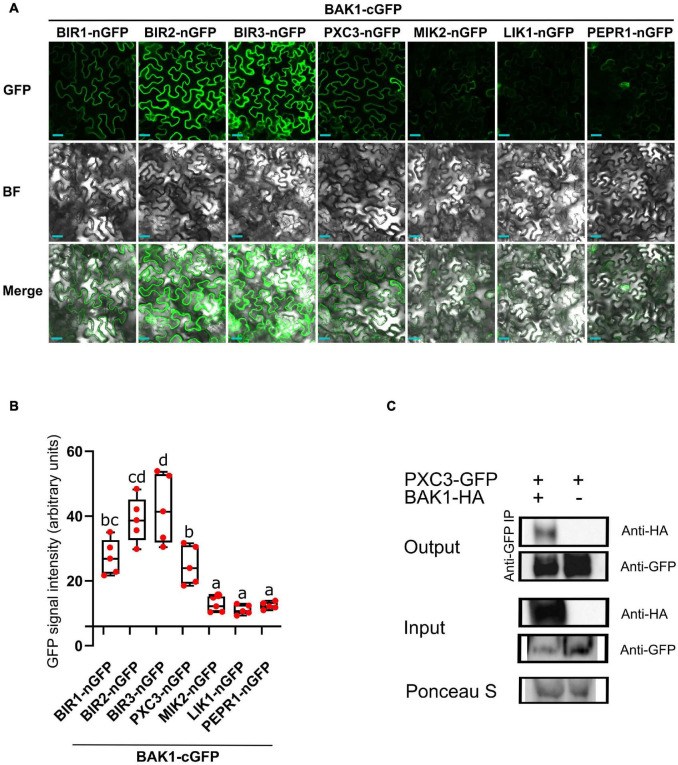
BAK1 interaction with identified LRR-RLKs. **(A)** BIFC experiments after transient expression in tobacco leaves of BAK1 and other receptors fused to the C-term or N-term halves of GFP, respectively. Scale bars represent 20 μm. This experiment was done three times with similar results. BF, bright field. **(B)** Quantification of the mean GFP signal in BIFC experiments. Individual data points represent values for different leaves (average of 5 images per interaction per leaf). A one-way ANOVA analysis was performed followed by Tukey’s test and the lowercase letters indicate statistical differences (*P* < 0.05). **(C)** Co-immunoprecipitation assay between BAK1 and PXC3 proteins. Protein extracts obtained from tobacco leaves infiltrated with Agrobacterium harboring *35Spro:BAK1-HA* and *35Spro:PXC3-GFP* were analyzed by western blot using anti-GFP and anti-HA antibodies.

Because known, as well as, unknown LRR-RLKs BAK1 interactors coeluted in all samples, we hypothesized that these are constitutive BAK1 interactors. To test this, we fused BAK1 to the C-terminal half (cGFP) and the six identified LRR-RLKs to the N-terminal half (nGFP) of the GFP protein, and performed BIFC analysis after transient expression in *N. benthamiana* leaves. As a negative control we used PEPR1, whose ectodomain has been shown to interact with BAK1 only upon addition of the AtPEP1 peptide ligand (Tang et al., 2015). BIFC assays revealed GFP signal for all tested combinations, which was associated to the plasma membrane, indicating that when fused to the GFP halves and combined, the tested pair of receptors still localized to the plasma membrane (Figure 1A). Quantification of the mean fluorescence intensity revealed a weak and patchy signal in the PEPR1-nGFP/BAK1-cGFP pair. This could be due to some PEPR1–BAK1 pre-complex formation without the ligand, basal expression of the PEP1 ligand in *N. benthamiana* leaves and/or some spontaneous association of the two GFP halves. Thus, we considered the PEPR1-nGFP/BAK1-cGFP as background and regarded as true interactions only those for which the measured signal was significantly higher than this value. In agreement with previous results, all three BIR1, BIR2, and BIR3 showed interaction with BAK1 by BIFC. Both, MIK2 and LIK1 receptors, showed weak GFP signal similar to the negative control. In contrast, PXC3 showed strong BIFC signal that could be indicative of true interaction with BAK1 (Figure 1B), which has not been previously reported.

To further validate the PXC3–BAK1 interaction, co-immunoprecipitation (Co-IP) assays were performed by co-infiltrating PXC3-GFP and BAK1-HA fusion proteins in tobacco leaves. Pull down with anti-GFP beads and western blot analyses showed that the BAK1-HA protein co-IPed with PXC3-GFP (Figure 1C). This confirms that PXC3 constitutively interacts with BAK1.

### Phloem Intercalated With Xylem-Correlated 3 Mutants Show Defects in Hypocotyl Vascular Development

**FIGURE 2:**
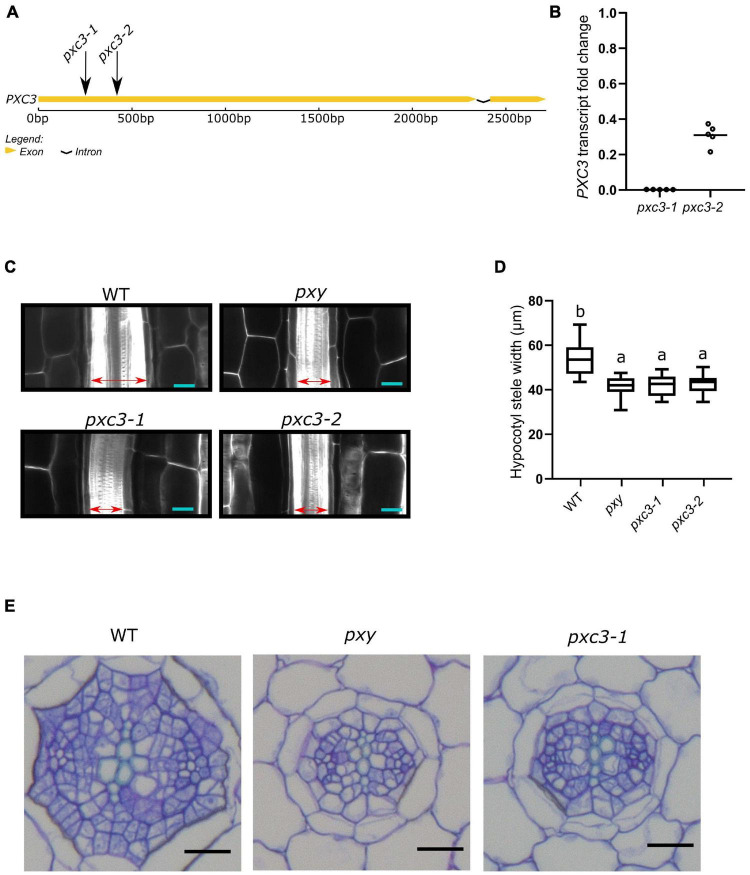
*pxc3* mutants have reduced stele in the hypocotyl of *Arabidopsis* seedlings. **(A)** Structure of *PXC3* gene. The arrows indicated the approximate position of the T-DNA insertion in *pxc3* mutants. **(B)** *PXC3* expression levels in *pxc3-1* and *pxc3-2* mutants compared to wild type determined by qPCR in 5 independent biological replicates (individual data points). Transcript fold change relative to wild type is shown. **(C)** Longitudinal confocal sections of wild type and mutant hypocotyls (7 DAG seedlings). The double-sided arrow indicates the stele width. Scale bars represent 20 μm. This experiment was done three times with similar results. **(D)** Quantification of the stele width in wild type and mutant hypocotyls (*n* ≥ 15). One-way ANOVA followed by Tukey’s test was performed, and the lowercase letters indicate statistical differences (*P* < 0.05). **(E)** Cross-section of wild type and mutant hypocotyls (7 DAG seedlings). Scale bars represent 20 μm.

PXC3 was previously shown to be co-expressed with TDR/PXY. In agreement with this, a *PXC3* transcriptional reporter (*PXC3pro::GUS*) displayed a signal in vascular strands of hypocotyl and roots in young seedlings (Wang et al., 2013). Nevertheless, no further studies were performed on PXC3 function. To gain insights into the PXC3 function we obtained two *lof* mutants, *pxc3-1* (SALK_121365) and *pxc3-2* (SALK_092805), both containing a T-DNA insertion in the coding sequence (Figure 2A). Expression analysis by qPCR showed that the *PXC3* mRNA levels were significantly reduced in both mutants compared to wild type (Figure 2B). We then analyzed longitudinal confocal sections of 7 DAG wild type and *pxc3* hypocotyls. The *pxy* mutant was also added as a control. The results show that the stele width in *pxy* is significantly reduced compared to wild type, in agreement with previous reports (Figures 2C,D; Hirakawa et al., 2010). Furthermore, similar defects were observed in both *pxc3* mutants (Figures 2C,D). The analysis of *pxc3* mutants suggests that PXC3 is involved in vascular development.

To further study the vascular defects in *pxc3* mutants, we performed hypocotyl cross-sections. As previously shown, the number of procambium cells in *pxy* mutants was reduced and the xylem and phloem were sometimes found in contact with each other (Fisher and Turner, 2007). Similarly, in the *pxc3-1* mutant, the number of procambium cells separating the xylem and the phloem poles was found to be reduced (Figure 2E). In summary, the defects observed in *pxc3* mutants are similar but milder than those in the *pxy* mutant.

### Phloem Intercalated With Xylem-Correlated 3 Positively Regulates the CLE41/TDIF–TDR/PXY Signaling Pathway During Vascular Development

**FIGURE 3:**
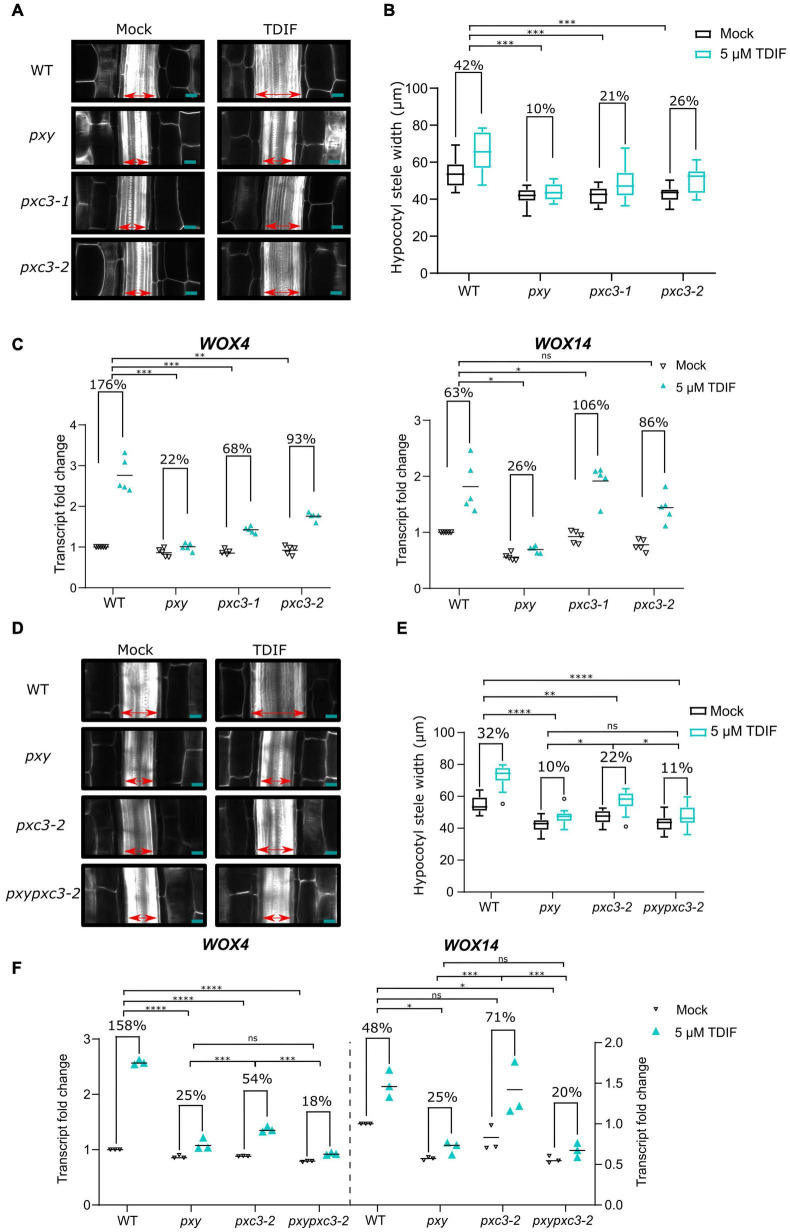
*pxc3* mutants have reduced sensitivity to TDIF peptide treatment. **(A)** Longitudinal confocal sections of wild type and mutant hypocotyls (7 DAG seedlings) treated or not with the TDIF (5 μM) peptide for 4 days as indicated. The double-sided arrow indicates the stele width. Scale bars represent 20 μm. **(B)** Quantification of the stele width (*n* ≥ 15). This experiment was done three times. In two out of three replicates, a significant interaction between treatment and genotype was found for *pxc3* mutants compared to wild type. Numbers indicate fold change in the mean stele width relative to the untreated sample for each genotype. A two-way ANOVA followed by a Dunnett’s test was performed and the asterisks indicate a significant genotype and treatment interaction compared to wild type (***P < 0.001). **(C)** *WOX4* and *WOX14* transcript levels after 8 h TDIF treatment of wild-type and mutant seedlings. Fold change relative to wild type (mock) is shown. Numbers indicate fold change in the mean value (line) relative to the untreated sample for each genotype (*n* = 5 independent replicates). A one-way ANOVA followed by a Tukey’s test was performed on Log2-transformed peptide/mock gene expression ratio, and the asterisks indicate a significant genotype and treatment interaction compared to wild type (*P < 0.05, ** *P* < 0.01, and ***P < 0.001, and ns indicates that no significant difference was found). **(D)** Longitudinal confocal sections of wild type and mutant hypocotyls (7 DAG seedlings) treated or not with the TDIF (5 μM) peptide for 4 days as indicated. The double-sided arrow indicates the stele width. Scale bars represent 20 μm. **(E)** Quantification of the stele width (*n* ≥ 15). This experiment was done two times. Numbers indicate fold change in the mean stele width relative to the untreated sample for each genotype. A two-way ANOVA followed by a Dunnett’s test was performed and the asterisks indicate a significant genotype and treatment interaction (*P < 0.05, **P < 0.01, and ****P < 0.0001, and ns indicates that no significant difference was found). **(F)** *WOX4* and *WOX14* transcript levels after 8 h TDIF treatment of wild-type and mutant seedlings. Fold change relative to wild type (mock) is shown. Numbers indicate fold change in the mean value (line) relative to the untreated sample for each genotype (*n* = 3 independent replicates). A one-way ANOVA followed by a Tukey’s test was performed on Log2-transformed peptide/mock gene expression ratio, and the asterisks indicate a significant genotype and treatment interaction (*P < 0.05, *** *P* < 0.001, and ***P < 0.001, and ns indicates that no significant difference was found).

*pxc3 lof* mutants showed a reduction in stele width and procambial cell number, similar to *pxy* mutant. In addition, we found that PXC3 interacts with BAK1 (Figures 1A–C). Since procambial proliferation is controlled by the CLE41/TDIF–TDR/PXY signaling, for which BAK1 is a coreceptor, we decided to investigate whether PXC3 is also part of this pathway. *CLE41* overexpression or synthetic TDIF peptide treatment leads to ectopic vascular cell proliferation and radial enlargement of the stele (Whitford et al., 2008; Etchells and Turner, 2010; Hirakawa et al., 2010). To determine whether PXC3 is involved in the CLE41/TDIF–TDR/PXY signaling pathway, we analyzed the stele width in hypocotyls of wild type, *pxy*, and the two *pxc3* mutants treated or not with the TDIF peptide. The result showed that, after the TDIF peptide treatment, the stele width was significantly enlarged in wild type while such an enlargement was not observed in the *pxy* mutant as previously reported (Hirakawa et al., 2010). In the two *pxc3* mutants, radial expansion of the stele was also observed upon TDIF peptide treatment, but the enlargement was significantly smaller than in the wild type in two out of three replicates, suggesting that *pxc3* mutants are less sensitive to TDIF peptide treatment (Figures 3A,B).

The CLE41/TDIF–TDR/PXY signaling pathway regulates procambial cell proliferation, through upregulation of *WOX4* and *WOX14* expression (Hirakawa et al., 2010; Etchells et al., 2013). To further confirm that PXC3 is involved in this signaling pathway, we analyzed the expression levels of *WOX4* and *WOX14* in wild type, *pxy*, and the *pxc3* mutants after TDIF peptide treatment for 8 h. As expected, TDIF peptide treatment triggered upregulation of *WOX4* and *WOX14* expression in wild-type seedlings while this effect was largely suppressed in *pxy* mutants (Figure 3C). Furthermore, the TDIF-induced increase in *WOX4* transcription was also partially suppressed in both the *pxc3* mutants, again confirming that the mutants are partially resistant to TDIF peptide treatment. However, opposite to *pxy*, *pxc3-1* and *pxc3-2* mutants did not suppress the induction of *WOX14* transcript by TDIF treatment. A slightly higher *WOX14* transcript induction was observed in *pxc3* mutants compared to the wild type (Figure 3C).

To determine whether PXY and PXC3 are part of the same or parallel pathway(s), we generated a *pxypxc3-2* double mutant and monitored hypocotyl width and *WOX4* and *WOX14* transcription in response to the TDIF peptide. The defects observed in *pxy* and *pxc3-2* mutants were not additive both, under mock conditions or peptide treatment, and the double mutant was indistinguishable from *pxy*, indicating that these two LRR-RLKs act most likely in the same genetic pathway (Figures 3D–F).

Wang et al. (2013) previously identified PXC1 to 3 to be co-expressed with PXY, which could indicate that not only PXC3 but also PXC1 and 2 are associated with the TIDIF–PXY pathway. Therefore, we finally investigated whether mutants in other *PXC* genes responded differently than wild type to the TDIF peptide. We could not recover mutants for *PXC1*, but we obtained two independent mutants for *PXC2* (also recently called Canalization-related Receptor-like kinase, CANAR) (Hajny et al., 2020). Interestingly, both *pxc2* mutants showed reduced response to TDIF, although in contrast to *pxc3* mutants, they did not show decreased stele width under mock conditions (Supplementary Figure 1).

### Phloem Intercalated With Xylem-Correlated 3 Loss-of-Function Does Not Affect Brassinosteroid Insensitive 1-Associated Receptor Kinase 1 Levels

**FIGURE 4:**
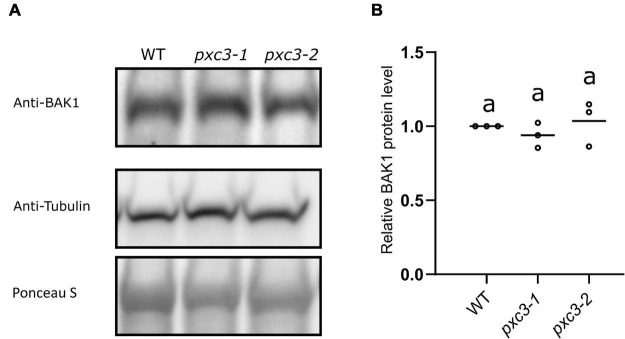
BAK1 protein levels in wild type and *pxc3* mutants. **(A)** Western blot analysis of BAK1 protein levels as detected by anti-BAK1 antibodies in wild type and *pxc3* mutant seedlings. Blots were reblotted with anti-tubulin in two out of three performed experiments, all showing similar results. **(B)** Quantification of BAK1 protein levels in wild type and *pxc3* mutants. For each experiment, BAK1 signal in the mutants was normalized by that in wild type. Individual data points for each experiment are shown. One-way ANOVA analysis was performed followed by Tukey’s test, and the lowercase letters indicate statistical differences (*P* < 0.05).

Our data showed that PXC3 constitutively interacts with BAK1 and seems to be involved in CLE41/TDIF–TDR/PXY signaling during vascular development. It could be that PXC3 positively regulates the CLE41/TDIF–TDR/PXY pathway by stabilizing BAK1. In addition to exerting a negative regulation on BRI1–BAK1 dimerization, BIR3 was reported to constitutively interact with and stabilize BAK1 in a ligand-independent manner. Accordingly, BAK1 levels are significantly reduced in *bir3* mutants compared to wild type (Imkampe et al., 2017). To test whether PXC3–BAK1 interaction results in BAK1 stabilization, we monitored BAK1 protein levels using anti-BAK1 antibodies in wild type and *pxc3* seedlings but no significant changes in BAK1 levels between *pxc3* mutants and wild type were detected (Figures 4A,B). Thus, the defects observed in *pxc3* mutants cannot be explained by decreased in BAK1 stability.

## Discussion

In this work, we found six LRR-RLKs that co-eluted with BAK1 in AP-MS experiments. Since three of these were recognized BAK1 interactors while the other three were not at the time we performed the experiment, we decided to verify the interaction with BAK1 by independent methods. Using BIFC, as well as Co-IP, we confirmed that PXC3 constitutively interacts with BAK1. Although we also detected some GFP signals in the BIFC experiment using MIK2 and LIK1, comparison to the PEPR1–BAK1 interaction did not reveal significant differences. However, PEPR1 and BAK1 may still weakly dimerize in tobacco leaves and if so, comparing to this stringent control will underestimate other positive BAK1 interactions. In agreement with PEPR1–BAK1 preassembly, we detected also PEPR1 peptides in our AP-MS experiment in *Arabidopsis* seedlings (Supplementary Table 1).

MIK2 belongs to subgroup XII of the LRR-RLKs and has been associated with different developmental processes in plants including plant reproduction, response to cell wall damage, salt stress, and pathogen-triggered immunity (PTI) (Julkowska et al., 2016; Wang et al., 2016; Van der Does et al., 2017; Coleman et al., 2021). A recent publication showed a weak association of MIK2 and BAK1 by co-IP and this interaction was enhanced by the addition of a *Fusarium* PTI-eliciting extract (Coleman et al., 2021). Furthermore, MIK2 was shown to perceive the phytocytokine SERINE RICH ENDOGENOUS PEPTIDE 12 (SCOOP12), and likely other peptides of the same family present in *Arabidopsis* and *Fusarium*, which also induces heterodimerization of MIK2 and BAK1 (Rhodes et al., 2021). It is thus likely, that the low MIK2–BAK1 association detected here represents pre-complexes that form but are not very abundant. The same may be true for LIK1. LIK1 associates to the LysM receptor-like kinase (LysM-RLK1/CERK1) involved in chitin perception and was proposed to negatively regulate chitin-induced innate immunity (Le et al., 2014). *lik1* mutants showed enhanced sensitivity not only to chitin but also to the bacterial-derived peptide flg22. Since BAK1 association to pattern recognition receptors such as FLS2 (the plant receptor sensing flg22) is necessary for PTI responses, LIK1 could act as a negative regulator of bacterial and fungal immunity by sequestering the coreceptor BAK1, as it has been shown for BIR proteins. However, this hypothesis, as well as the LIK1–BAK1 interaction, still has to be confirmed by independent approaches.

In higher plants, the vasculature is essential for the movement of resources, like water and sugars, throughout the plant body. CLE41/TDIF–TDR/PXY signaling controls vascular development by regulating vascular cell division, bundle organization, and xylem differentiation (Fisher and Turner, 2007; Etchells and Turner, 2010; Fukuda and Hardtke, 2020). This pathway also uses BAK1 as coreceptor (Zhang et al., 2016). Here, we found PXC3 to interact with BAK1 and *pxc3* mutants show reduced stele width compared to wild type, corresponding to a decreased number of procambial cells. Besides, *pxc3* mutants are less sensitive to TDIF peptide treatments, as monitored by the TDIF-induced stele enlargement and *WOX4* upregulation. Altogether, our data suggest that PXC3 controls procambial division as part of the CLE41/TDIF–TDR/PXY signaling pathway.

We could see two possible scenarios for PXC3 function. First, PXC3 could positively regulate the PXY and BAK1 interaction as it has been previously shown for other ligand-free LRR-RLK interactions. For example, the LRR-RLK FIR, as well as IOS1, interact with FLS2 and BAK1 constitutively and promotes FLS2–BAK1 complex formation in the presence of the flg22 ligand (Yeh et al., 2016; Smakowska-Luzan et al., 2018). Another possibility is that PXC3 binds and senses the TDIF peptide in parallel to PXY to regulate cell division. PXC3 belongs to subgroup XI of LRR-RLKs and its ectodomain contains 18 LRRs, thus, it could function as a ligand-sensing receptor. An alternative receptor for the TDIF peptide has been proposed since the *pxywox4*, but not *pxywox14*, double mutant show enhanced division defects compared to the *pxy* mutant alone (Hirakawa et al., 2010; Etchells et al., 2013). Because the vascular expansion caused by overexpressing *CLE41* is to a large extent suppressed in *pxy*, this alternative receptor signaling would only have a minor contribution to vascular cell division (Etchells and Turner, 2010; Etchells et al., 2013). In agreement with this model, we observed a decrease in TDIF-induced *WOX4* but not *WOX14* transcript in *pxc3* mutants compared to wild type, indicating that PXY and PXC3 redundantly regulate TDIF-induced *WOX4*, but not *WOX14* levels. A similar reduction in stele width and procambial cell number of *pxy* and *pxc3* mutants (although reductions in *pxc3* are milder) also correlates with this proposition. The mild increase in *WOX14* transcription in *pxc3* mutants could be part of a feedback/compensatory mechanism. Nevertheless, the constitutive PXC3–BAK1 interaction argues against this hypothesis, since receptor–coreceptor interactions are most often induced by the ligand (Hohmann et al., 2017). The generation of a double *pxypxc3-2* mutant further confirmed that PXC3 and PXY are likely part of the same genetic pathway and it is most probable that PXC3 has a regulatory function (i.e., in PXY–BAK1 complex formation) as has been shown for other BAK1-dependent receptor complexes (Yeh et al., 2016; Smakowska-Luzan et al., 2018).

The reported *PXC3* expression (Wang et al., 2013) comprises vascular tissues in both roots and shoots, which can be confirmed from public microarray compendia^1^. Based on this, it is possible that PXC3 has a similar function in procambial cell proliferation in the root as in the hypocotyl. However, since the TDIF–PXY/TDR pathway has been mostly characterized in shoot tissues, the function of PXC3 in root vascular development should be investigated in future studies. In addition, the *PXC3* expression in shoot tissues expands to non-vascular cells (Wang et al., 2013). However, we have to point out that we did not observe any other obvious developmental defects at the vegetative or reproductive stage, nor did we find a defect in other hypocotyl cell layers in transversal sections (i.e., epidermis or cortex cell number/size). Nevertheless, we cannot rule out a less significant function of PXC3 in other shoot cells/tissues or under certain conditions (i.e., stress). Thus, targeted investigation of other cells or plant processes not considered in this study may reveal additional functions of the PXC3-BAK1 interaction in future studies.

In the original report where PXC3 was identified by co-expression analysis, two other LRR-RLKs, PXC1, and PXC2 were identified as well, although their involvement in the CLE41/TDIF–TDR/PXY pathway was not further investigated (Wang et al., 2013). Analysis of *pxc1* mutants revealed reduced lignin content in inflorescence stems, and thus, PXC1 was associated with secondary wall deposition in fiber cells (Wang et al., 2013). Recently, PXC2/CANAR was associated with the regeneration of vascular strands after wounding (Hajny et al., 2020). A preprint article also shows that *pxc2* mutants enhanced the defects in *irk4*, a mutant of another LRR-RLK, in the root apical meristem, which results in ectopic anticlinal divisions in endodermal and stele cells. The *pxc2irk4* double mutants display even more enlarged stele than single *irk4* mutants, suggesting a function for PXC2 in restricting anticlinal divisions in endodermis and stele cells in the root (Goff and Van Norman, 2021). Here we showed that *pxc2* mutants also responded less to the TDIF peptide, indicating that the originally identified *PXC* genes are potential regulators of the CLE41/TDIF–TDR/PXY pathway. Multiple interactions and interplay between different LRR-RLKs have in the last years revealed to be highly complex (Smakowska-Luzan et al., 2018). Thus, the function of PXC1 to 3 in the CLE41/TDIF–TDR/PXY, as well as, other pathways needs to be fully characterized in the future.

## Data Availability Statement

The original contributions presented in the study are publicly available. This data can be found here: ProteomeXchange *via* the PRIDE database with accession number: PXD026487.

## Author Contributions

AF performed AP-MS experiments. KX, AF, and LN confirmed receptor interactions. KX, JJ, and MN performed and analyzed hypocotyl cross-sections. KX analyzed the stele width and performed qPCR experiments. KX and JJ performed the statistical analysis. KX, AF, and TB wrote the manuscript with input from all other authors. AF and TB provided supervision on the experiments and the analysis of the results. All authors contributed to the article and approved the submitted version.

## Conflict of Interest

The authors declare that the research was conducted in the absence of any commercial or financial relationships that could be construed as a potential conflict of interest.

## Publisher’s Note

All claims expressed in this article are solely those of the authors and do not necessarily represent those of their affiliated organizations, or those of the publisher, the editors and the reviewers. Any product that may be evaluated in this article, or claim that may be made by its manufacturer, is not guaranteed or endorsed by the publisher.

## Footnotes

1
http://bar.utoronto.ca/eplant/

## Supplementary Material

Supplementary Figure 1 *pxc2* Mutants have reduced sensitivity to TDIF peptide treatment. **(A)** Longitudinal confocal sections of wild type and mutant hypocotyls (7 DAG seedlings) treated or not with the TDIF (5 μM) peptide for 4 days as indicated. The double-sided arrow indicates the stele width. Scale bars represent 20 μm. **(B)** Quantification of the stele width (*n* ≥ 15). This experiment was done two times. Numbers indicate fold change in the mean stele width relative to the untreated sample for each genotype. A two-way ANOVA followed by a Dunnett’s test was performed and the asterisks indicate a significant genotype and treatment interaction compared to wild type (^***^*P* < 0.001 and ^****^*P* < 0.0001). **(C)** *WOX4* and *WOX14* transcript levels after 8 h TDIF treatment of wild type and mutant seedlings. Fold change relative to wild type (mock) is shown. Numbers indicate fold change in the mean value (line) relative to the untreated sample for each genotype (*n* = 3 independent replicates). A one-way ANOVA followed by a Tukey’s test was performed on Log2-transformed peptide/mock gene expression ratio, and the asterisks indicate a significant genotype and treatment interaction compared to wild type (**P* < 0.05, ^***^*P* < 0.001, and ^****^*P* < 0.0001, and ns indicates that no significant difference was found).

Supplementary Table 1 Protein groups obtained after MaxQuant analysis of mock and estradiol-treated *BAK1pro:BAK1-GFP/bak1-4/iGLV6.* Protein groups are ordered by the intensity-based absolute quantification (iBAQ) values.

Supplementary Table 2 Primers used to monitor transcript levels by qPCR of *PXC3*, *WOX4*, and *WOX14*.

## References

[B1] Abas L. Luschnig C. (2010). Maximum yields of microsomal-type membranes from small amounts of plant material without requiring ultracentrifugation. *Anal. Biochem.* 401 217–227. 10.1016/j.ab.2010.02.030 20193653 PMC3685806

[B2] Beeckman T. Viane R. (2000). Embedding thin plant specimens for oriented sectioning. *Biotech. Histochem.* 75 23–26. 10.3109/10520290009047981 10810979

[B3] Boruc J. Van den Daele H. Hollunder J. Rombauts S. Mylle E. Hilson P. (2010). Functional modules in the *Arabidopsis* core cell cycle binary protein-protein interaction network. *Plant Cell* 22 1264–1280. 10.1105/tpc.109.073635 20407024 PMC2879739

[B4] Chinchilla D. Bauer Z. Regenass M. Boller T. Felix G. (2006). The Arabidopsis receptor kinase FLS2 binds flg22 and determines the specificity of flagellin perception. *Plant Cell* 18 465–476. 10.1105/tpc.105.036574 16377758 PMC1356552

[B5] Chinchilla D. Zipfel C. Robatzek S. Kemmerling B. Nurnberger T. Jones J. D. (2007). A flagellin-induced complex of the receptor FLS2 and BAK1 initiates plant defence. *Nature* 448 497–500. 10.1038/nature05999 17625569

[B6] Coleman A. D. Maroschek J. Raasch L. Takken F. L. W. Ranf S. Huckelhoven R. (2021). The Arabidopsis leucine-rich repeat receptor-like kinase MIK2 is a crucial component of early immune responses to a fungal-derived elicitor. *New Phytol.* 229 3453–3466. 33253435 10.1111/nph.17122

[B7] Couto D. Zipfel C. (2016). Regulation of pattern recognition receptor signalling in plants. *Nat. Rev. Immunol.* 16 537–552. 10.1038/nri.2016.77 27477127

[B8] De Smet I. Chaerle P. Vanneste S. De Rycke R. Inze D. Beeckman T. (2004). An easy and versatile embedding method for transverse sections. *J. Microsc.* 213 76–80. 10.1111/j.1365-2818.2004.01269.x 14678515

[B9] De Smet I. Voss U. Jurgens G. Beeckman T. (2009). Receptor-like kinases shape the plant. *Nat. Cell Biol.* 11 1166–1173. 10.1038/ncb1009-1166 19794500

[B10] Etchells J. P. Turner S. R. (2010). The PXY-CLE41 receptor ligand pair defines a multifunctional pathway that controls the rate and orientation of vascular cell division. *Development* 137 767–774. 10.1242/dev.044941 20147378

[B11] Etchells J. P. Provost C. M. Mishra L. Turner S. R. (2013). WOX4 and WOX14 act downstream of the PXY receptor kinase to regulate plant vascular proliferation independently of any role in vascular organisation. *Development* 140 2224–2234. 10.1242/dev.091314 23578929 PMC3912870

[B12] Fernandez A. I. Vangheluwe N. Xu K. Jourquin J. Claus L. A. N. Morales-Herrera S. (2020). GOLVEN peptide signalling through RGI receptors and MPK6 restricts asymmetric cell division during lateral root initiation. *Nat. Plants* 6 533–543. 10.1038/s41477-020-0645-z 32393883

[B13] Fernandez A. Drozdzecki A. Hoogewijs K. Vassileva V. Madder A. Beeckman T. (2015). The GLV6/RGF8/CLEL2 peptide regulates early pericycle divisions during lateral root initiation. *J. Exp. Bot.* 66 5245–5256. 10.1093/jxb/erv329 26163695 PMC4526922

[B14] Fisher K. Turner S. (2007). PXY, a receptor-like kinase essential for maintaining polarity during plant vascular-tissue development. *Curr. Biol.* 17 1061–1066. 10.1016/j.cub.2007.05.049 17570668

[B15] Fukuda H. Hardtke C. S. (2020). Peptide signaling pathways in vascular differentiation([OPEN]). *Plant Physiol.* 182 1636–1644. 10.1104/pp.19.01259 31796560 PMC7140915

[B16] Gao M. Wang X. Wang D. Xu F. Ding X. Zhang Z. (2009). Regulation of cell death and innate immunity by two receptor-like kinases in Arabidopsis. *Cell Host Microbe* 6 34–44. 10.1016/j.chom.2009.05.019 19616764

[B17] Goff J. Van Norman J. M. (2021). *Polarly Localized Receptor-Like Kinases PXC2 and IRK Act Redundantly During Arabidopsis Root Development In The Radial Axis. biorxiv* [Preprint]. Available online at: https://www.biorxiv.org/content/10.1101/2021.02.11.429611v1 [Accessed Februrary 21, 2021].

[B18] Gomez-Gomez L. Boller T. (2000). FLS2: an LRR receptor-like kinase involved in the perception of the bacterial elicitor flagellin in *Arabidopsis*. *Mol. Cell* 5 1003–1011. 10.1016/s1097-2765(00)80265-8 10911994

[B19] Hajny J. Prat T. Rydza N. Rodriguez L. Tan S. Verstraeten I. (2020). Receptor kinase module targets PIN-dependent auxin transport during canalization. *Science* 370 550–557. 10.1126/science.aba3178 33122378 PMC7116426

[B20] Halter T. Imkampe J. Mazzotta S. Wierzba M. Postel S. Bucherl C. (2014). The leucine-rich repeat receptor kinase BIR2 Is a negative regulator of BAK1 in plant immunity. *Curr. Biol.* 24 134–143. 10.1016/j.cub.2013.11.047 24388849

[B21] He Y. Zhou J. Shan L. Meng X. (2018). Plant cell surface receptor-mediated signaling – a common theme amid diversity. *J. Cell Sci.* 131:jcs209353. 10.1242/jcs.209353 29378836 PMC6518216

[B22] Hirakawa Y. Kondo Y. Fukuda H. (2010). TDIF peptide signaling regulates vascular stem cell proliferation *via* the WOX4 homeobox gene in *Arabidopsis*. *Plant Cell* 22 2618–2629. 10.1105/tpc.110.076083 20729381 PMC2947162

[B23] Hirakawa Y. Shinohara H. Kondo Y. Inoue A. Nakanomyo I. Ogawa M. (2008). Non-cell-autonomous control of vascular stem cell fate by a CLE peptide/receptor system. *Proc. Natl. Acad. Sci. U.S.A.* 105 15208–15213. 10.1073/pnas.0808444105 18812507 PMC2567516

[B24] Hohmann U. Lau K. Hothorn M. (2017). The structural basis of ligand perception and signal activation by receptor kinases. *Annu. Rev. Plant Biol.* 68 109–137. 10.1146/annurev-arplant-042916-040957 28125280

[B25] Imkampe J. Halter T. Huang S. Schulze S. Mazzotta S. Schmidt N. (2017). The *Arabidopsis* leucine-rich repeat receptor kinase BIR3 negatively regulates BAK1 receptor complex formation and stabilizes BAK1. *Plant Cell* 29 2285–2303. 10.1105/tpc.17.00376 28842532 PMC5635992

[B26] Ito Y. Nakanomyo I. Motose H. Iwamoto K. Sawa S. Dohmae N. (2006). Dodeca-CLE peptides as suppressors of plant stem cell differentiation. *Science* 313 842–845. 10.1126/science.1128436 16902140

[B27] Julkowska M. M. Klei K. Fokkens L. Haring M. A. Schranz M. E. Testerink C. (2016). Natural variation in rosette size under salt stress conditions corresponds to developmental differences between *Arabidopsis* accessions and allelic variation in the LRR-KISS gene. *J. Exp. Bot.* 67 2127–2138. 10.1093/jxb/erw015 26873976 PMC4809279

[B28] Karimi M. Bleys A. Vanderhaeghen R. Hilson P. (2007). Building blocks for plant gene assembly. *Plant Physiol.* 145 1183–1191. 10.1104/pp.107.110411 17965171 PMC2151724

[B29] Kurihara D. Mizuta Y. Sato Y. Higashiyama T. (2015). ClearSee: a rapid optical clearing reagent for whole-plant fluorescence imaging. *Development* 142 4168–4179. 10.1242/dev.127613 26493404 PMC4712841

[B30] Ladwig F. Dahlke R. I. Stuhrwohldt N. Hartmann J. Harter K. Sauter M. (2015). Phytosulfokine regulates growth in *Arabidopsis* through a response module at the plasma membrane that includes CYCLIC NUCLEOTIDE-GATED CHANNEL17, H+-ATPase, and BAK1. *Plant Cell* 27 1718–1729. 10.1105/tpc.15.00306 26071421 PMC4498212

[B31] Le M. H. Cao Y. Zhang X. C. Stacey G. (2014). LIK1, a CERK1-interacting kinase, regulates plant immune responses in *Arabidopsis*. *PLoS One* 9:e102245. 10.1371/journal.pone.0102245 25036661 PMC4103824

[B32] Li B. Ferreira M. A. Huang M. Camargos L. F. Yu X. Teixeira R. M. (2019). The receptor-like kinase NIK1 targets FLS2/BAK1 immune complex and inversely modulates antiviral and antibacterial immunity. *Nat. Commun.* 10:4996. 10.1038/s41467-019-12847-6 31676803 PMC6825196

[B33] Li J. Chory J. (1997). A putative leucine-rich repeat receptor kinase involved in brassinosteroid signal transduction. *Cell* 90 929–938. 10.1016/s0092-8674(00)80357-8 9298904

[B34] Li J. Wen J. Lease K. A. Doke J. T. Tax F. E. Walker J. C. (2002). BAK1, an Arabidopsis LRR receptor-like protein kinase, interacts with BRI1 and modulates brassinosteroid signaling. *Cell* 110 213–222. 10.1016/s0092-8674(02)00812-7 12150929

[B35] Ma X. Xu G. He P. Shan L. (2016). SERKing coreceptors for receptors. *Trends Plant Sci.* 21 1017–1033. 10.1016/j.tplants.2016.08.014 27660030

[B36] Matsubayashi Y. Ogawa M. Morita A. Sakagami Y. (2002). An LRR receptor kinase involved in perception of a peptide plant hormone, phytosulfokine. *Science* 296 1470–1472. 10.1126/science.1069607 12029134

[B37] Meng X. Chen X. Mang H. Liu C. Yu X. Gao X. (2015). Differential function of *Arabidopsis* SERK family receptor-like kinases in stomatal patterning. *Curr. Biol.* 25 2361–2372. 10.1016/j.cub.2015.07.068 26320950 PMC4714584

[B38] Meng X. Zhou J. Tang J. Li B. de Oliveira M. V. V. Chai J. (2016). Ligand-induced receptor-like kinase complex regulates floral organ abscission in *Arabidopsis*. *Cell Rep.* 14 1330–1338. 10.1016/j.celrep.2016.01.023 26854226 PMC4758877

[B39] Ntoukakis V. Schwessinger B. Segonzac C. Zipfel C. (2011). Cautionary notes on the use of C-terminal BAK1 fusion proteins for functional studies. *Plant Cell* 23 3871–3878. 10.1105/tpc.111.090779 22129600 PMC3246322

[B40] Ou Y. Kui H. Li J. (2021). Receptor-like kinases in root development: current progress and future directions. *Mol. Plant* 14 166–185. 10.1016/j.molp.2020.12.004 33316466

[B41] Ou Y. Lu X. Zi Q. Xun Q. Zhang J. Wu Y. (2016). RGF1 INSENSITIVE 1 to 5, a group of LRR receptor-like kinases, are essential for the perception of root meristem growth factor 1 in *Arabidopsis thaliana*. *Cell Res.* 26 686–698. 10.1038/cr.2016.63 27229312 PMC4897188

[B42] Rhodes J. Yang H. Moussu S. Boutrot F. Santiago J. Zipfel C. (2021). Perception of a divergent family of phytocytokines by the *Arabidopsis* receptor kinase MIK2. *Nat. Commun.* 12:705. 10.1038/s41467-021-20932-y 33514716 PMC7846792

[B43] Roux M. Schwessinger B. Albrecht C. Chinchilla D. Jones A. Holton N. (2011). The *Arabidopsis* leucine-rich repeat receptor-like kinases BAK1/SERK3 and BKK1/SERK4 are required for innate immunity to hemibiotrophic and biotrophic pathogens. *Plant Cell* 23 2440–2455. 10.1105/tpc.111.084301 21693696 PMC3160018

[B44] Santiago J. Brandt B. Wildhagen M. Hohmann U. Hothorn L. A. Butenko M. A. (2016). Mechanistic insight into a peptide hormone signaling complex mediating floral organ abscission. *Elife* 5:e15075. 10.7554/eLife.15075 27058169 PMC4848090

[B45] Scholthof H. B. (2006). Timeline – the tombusvirus-encoded P19: from irrelevance to elegance. *Nat. Rev. Microbiol.* 4 405–411. 10.1038/nrmicro1395 16518419

[B46] Shiu S. H. Bleecker A. B. (2001). Receptor-like kinases from *Arabidopsis* form a monophyletic gene family related to animal receptor kinases. *Proc. Natl. Acad. Sci. U.S.A.* 98 10763–10768. 10.1073/pnas.181141598 11526204 PMC58549

[B47] Shiu S. H. Bleecker A. B. (2003). Expansion of the receptor-like kinase/Pelle gene family and receptor-like proteins in *Arabidopsis*. *Plant Physiol.* 132 530–543. 10.1104/pp.103.021964 12805585 PMC166995

[B48] Smaczniak C. Li N. Boeren S. America T. van Dongen W. Goerdayal S. S. (2012). Proteomics-based identification of low-abundance signaling and regulatory protein complexes in native plant tissues. *Nat. Protoc.* 7:2144–2158. 10.1038/nprot.2012.129 23196971

[B49] Smakowska-Luzan E. Mott G. A. Parys K. Stegmann M. Howton T. C. Layeghifard M. (2018). An extracellular network of *Arabidopsis* leucine-rich repeat receptor kinases. *Nature* 553 342–346. 10.1038/nature25184 29320478 PMC6485605

[B50] Smit M. E. McGregor S. R. Sun H. Gough C. Bagman A. M. Soyars C. L. (2020). A PXY-mediated transcriptional network integrates signaling mechanisms to control vascular development in *Arabidopsis*. *Plant Cell* 32 319–335. 10.1105/tpc.19.00562 31806676 PMC7008486

[B51] Song W. Liu L. Wang J. Wu Z. Zhang H. Tang J. (2016). Signature motif-guided identification of receptors for peptide hormones essential for root meristem growth. *Cell Res.* 26 674–685. 10.1038/cr.2016.62 27229311 PMC4897187

[B52] Tang J. Han Z. Sun Y. Zhang H. Gong X. Chai J. (2015). Structural basis for recognition of an endogenous peptide by the plant receptor kinase PEPR1. *Cell Res.* 25 110–120. 10.1038/cr.2014.161 25475059 PMC4650589

[B53] van der Burgh A. M. Joosten M. (2019). Plant immunity: thinking outside and inside the box. *Trends Plant Sci.* 24 587–601. 10.1016/j.tplants.2019.04.009 31171472

[B54] Van der Does D. Boutrot F. Engelsdorf T. Rhodes J. McKenna J. F. Vernhettes S. (2017). The Arabidopsis leucine-rich repeat receptor kinase MIK2/LRR-KISS connects cell wall integrity sensing, root growth and response to abiotic and biotic stresses. *PLoS Genet.* 13:e1006832. 10.1371/journal.pgen.1006832 28604776 PMC5484538

[B55] Van Leene J. Stals H. Eeckhout D. Persiau G. Van De Slijke E. Van Isterdael G. (2007). A tandem affinity purification-based technology platform to study the cell cycle interactome in *Arabidopsis thaliana*. *Mol. Cell Proteomics* 6 1226–1238. 10.1074/mcp.M700078-MCP200 17426018

[B56] Wang J. Kucukoglu M. Zhang L. Chen P. Decker D. Nilsson O. (2013). The Arabidopsis LRR-RLK, PXC1, is a regulator of secondary wall formation correlated with the TDIF-PXY/TDR-WOX4 signaling pathway. *BMC Plant Biol.* 13:94. 10.1186/1471-2229-13-94 23815750 PMC3716795

[B57] Wang J. Li H. Han Z. Zhang H. Wang T. Lin G. (2015). Allosteric receptor activation by the plant peptide hormone phytosulfokine. *Nature* 525 265–268. 10.1038/nature14858 26308901

[B58] Wang T. Liang L. Xue Y. Jia P. F. Chen W. Zhang M. X. (2016). A receptor heteromer mediates the male perception of female attractants in plants. *Nature* 531 241–244. 10.1038/nature16975 26863186

[B59] Wendrich J. R. Boeren S. Moller B. K. Weijers D. De Rybel B. (2017). In vivo identification of plant protein complexes using IP-MS/MS. *Methods Mol. Biol.* 1497 147–158. 10.1007/978-1-4939-6469-7_14 27864765

[B60] Whitford R. Fernandez A. De Groodt R. Ortega E. Hilson P. (2008). Plant CLE peptides from two distinct functional classes synergistically induce division of vascular cells. *Proc. Natl. Acad. Sci. U.S. A.* 105 18625–18630. 10.1073/pnas.0809395105 19011104 PMC2587568

[B61] Xi L. Wu X. N. Gilbert M. Schulze W. X. (2019). Classification and interactions of LRR receptors and Co-receptors within the *Arabidopsis* plasma membrane – an overview. *Front. Plant Sci.* 10:472. 10.3389/fpls.2019.00472 31057579 PMC6477698

[B62] Yeh Y. H. Panzeri D. Kadota Y. Huang Y. C. Huang P. Y. Tao C. N. (2016). The *Arabidopsis* malectin-Like/LRR-RLK IOS1 is critical for BAK1-dependent and BAK1-independent pattern-triggered immunity. *Plant Cell* 28 1701–1721. 10.1105/tpc.16.00313 27317676 PMC5077175

[B63] Zhang H. Q. Lin X. Y. Han Z. F. Wang J. Z. Qu L. J. Chai J. J. (2016). SERK family receptor-like kinases function as co-receptors with PXY for plant vascular development. *Mol. Plant* 9 1406–1414. 27449136 10.1016/j.molp.2016.07.004

