# Supplementary figures and images for: The Phloem Intercalated With Xylem-Correlated 3 Receptor-Like Kinase Constitutively Interacts With Brassinosteroid Insensitive 1-Associated Receptor Kinase 1 and Is Involved in Vascular Development in Arabidopsis
Source: Front Plant Sci. 2022 Jan 11;12:706633. doi: 10.3389/fpls.2021.706633 (PMC8786740; doi:10.3389/fpls.2021.706633)

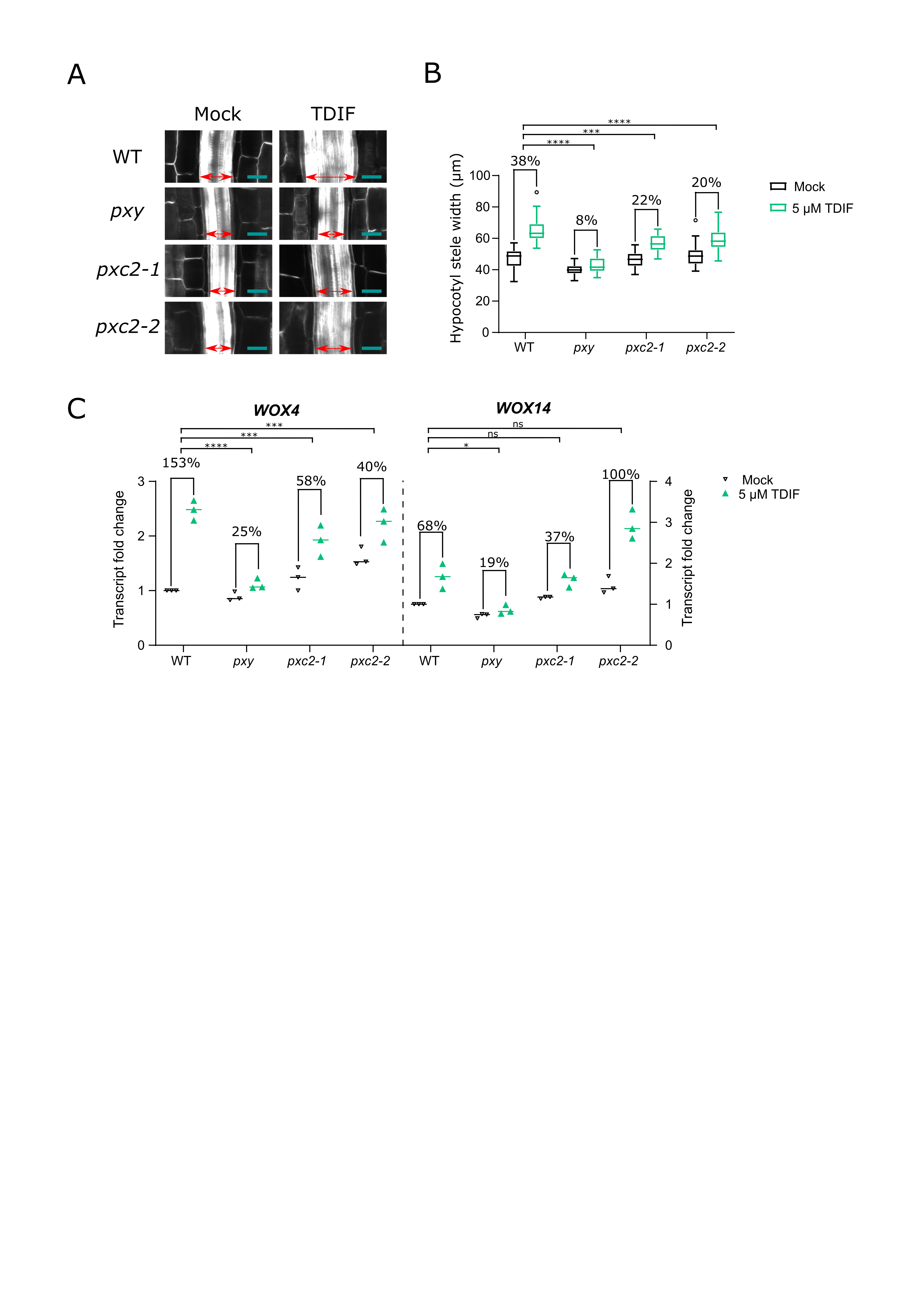

Supplement: Supplementary Figure 1 — pxc2 Mutants have reduced sensitivity to TDIF peptide treatment. (A) Longitudinal confocal sections of wild type and mutant hypocotyls (7 DAG seedlings) treated or not with the TDIF (5 μM) peptide for 4 days as indicated. The double-sided arrow indicates the stele width. Scale bars represent 20 μm. (B) Quantification of the stele width (n ≥ 15). This experiment was done two times. Numbers indicate fold change in the mean stele width relative to the untreated sample for each genotype. A two-way ANOVA followed by a Dunnett’s test was performed and the asterisks indicate a significant genotype and treatment interaction compared to wild type (***P < 0.001 and ****P < 0.0001). (C) WOX4 and WOX14 transcript levels after 8 h TDIF treatment of wild type and mutant seedlings. Fold change relative to wild type (mock) is shown. Numbers indicate fold change in the mean value (line) relative to the untreated sample for each genotype (n = 3 independent replicates). A one-way ANOVA followed by a Tukey’s test was performed on Log2-transformed peptide/mock gene expression ratio, and the asterisks indicate a significant genotype and treatment interaction compared to wild type (*P < 0.05, ***P < 0.001, and ****P < 0.0001, and ns indicates that no significant difference was found). [file Image_1.PNG]
